# Change in mammographic density as a potential predictor of cancer recurrence after breast conservation surgery and adjuvant endocrine therapy: results of the MEDICI study

**DOI:** 10.1186/s13058-026-02239-2

**Published:** 2026-02-19

**Authors:** Sarah L Savaridas, Andrea Marshall, Kulsam Ali, Susan M Astley, Andrew J Evans, Mark Halling-Brown, Sarah Vinnicombe, Violet R Warwick, Patsy Whelehan, Sonya Drummond, Raja Ebsim, Nuala A Healy, Jonathan Nash, Elizabeth Muscat, Sreenivas Muthyala, Ashwini Sharma, Marianna Telesca, Janet Dunn

**Affiliations:** 1 University of Dundee, Dundee, Scotland; 2 NHS Tayside, Dundee, Scotland; 3 Warwick Clinical Trials Unit, University of Warwick, Coventry, England; 4 Division of Informatics, Imaging and Data science, The University of Manchester, Manchester, England; 5 Royal Derby Hospital, Derby, England; 6 Royal Surrey NHS Foundation Trust, Guildford, UK; 7 Thirlestaine Breast Centre, Gloucestershire NHS Foundation Trust, Gloucester, England; 8 University of St Andrews, St Andrews, Scotland; 9 Great Western Hospitals NHS Foundation Trust, Swindon, UK; 10 Department of Radiology, Royal College of Surgeons in Ireland, Dublin, Ireland; 11 Beaumount Breast Centre, Beaumont Hospital, Dublin, Ireland; 12 BreastCheck Southern Unit, Cork, Ireland; 13 St George’s University Hospitals NHS Foundation Trust, London, England; 14 NHS Lothian, Edinburgh, Scotland; 15 Worcestershire Acute Hospitals NHS Trust, Worcester, England

**Keywords:** Mammographic density, Adjuvant endocrine therapy, Breast cancer specific survival, Metastasis free survival, Disease free survival, Breast density, Tamoxifen, Aromatase inhibitors

## Abstract

**Background:**

Oestrogen-receptor positive breast cancer patients are typically treated with adjuvant endocrine therapy (AET), some develop AET resistance. Previous research suggests mammographic density (MD) may represent an imaging biomarker, with fewer local or distant recurrences occurring with decreasing MD. We investigate whether reduction in MD after 1 and/or 3 years is associated with improved breast cancer specific survival (BCSS), metastasis-free survival (MFS) or disease-free survival (DFS).

**Methods:**

This retrospective cohort study was generated from a Mammo-50 trial subset. Participants taking AET (cases) and controls were included. MD was assessed in the AET group using a 0–100% visual analogue scale (VAS). Readers scored mammograms at diagnosis, 1 year and 3 years post-surgery. A decrease in MD was defined as ≥10% reduction from diagnosis. A second reader reviewed paired mammograms and assessed whether there had been a temporal change in MD.

**Results:**

Data from 1364 cases and 367 controls were included. Median VAS MD was approximately 30% for cases and controls at all time-points; 20% showed decreased MD at 1 year and 21% at 3 years for both cases and controls. Of the AET group, 23 died from breast cancer and 33 developed metastases during follow-up (median 8.7 years post-surgery). The 5-year BCSS rate was 99.6% (95%CI:97.4-99.9) versus 98.3% (95%CI:97.2-98.9) for those with and without a ≥10% reduction in MD at 1 year, *p*=0.35. The 5-year MFS rate for those with and without a ≥10% reduction in MD at 1 year was 94.2% (95%CI:90.7-96.4) versus 93.6% (95%CI:92.0-95.0) respectively; *p*=0.47. The 5-year DFS rate for those with a ≥10% reduction in MD at 1year was 92.4% (95% CI:88.5-94.9) versus 92.6% (95%CI:90.8-94.0); *p*= 0.47. Similar results for BCSS, MFS and DFS were seen for those with and without a ≥10% reduction in MD at 3 years and for those assessed as having a definite reduction in MD compared to those who had not at both 1 and 3 years.

**Conclusion:**

Reduction in MD had no significant association with rates of BCSS, MFS or DFS. Change in MD was not shown to be a useful prognostic indicator in women over 50 years, treated with AET.

## Background

Adjuvant endocrine therapy (AET) is routinely used to treat patients with oestrogen receptor (ER) positive breast cancer. However, AET can be poorly tolerated, and many ER positive patients eventually develop resistance to an endocrine intervention [1, 2]. AET resistance is when cancer cells no longer respond to AET leading to relapse and progression of disease; local or systemic recurrence. Whilst it is currently not possible to categorically identify cases of AET resistance, from a clinical perspective, if a patient were to develop a new or recurrent breast cancer whilst taking AET, resistance would be assumed and the AET changed.

Dense breast tissue refers to the proportion of fibroglandular tissue as opposed to fatty tissue within the breast. Not only does increased mammographic density (MD) reduce the sensitivity of mammography due to obscuration of disease, it is a strong and well-established independent risk factor for breast cancer [3, 4]. Extensive research has been conducted using visual assessment of area-based MD on images, including a method of recording percentage density by area using Visual Analogue Scales (VAS). The relationship with breast cancer risk has been demonstrated in many studies [5, 6], with further research showing that it adds value to established breast cancer risk models [7].

The relationship between MD, endocrine therapy and outcomes was first demonstrated by the International Breast Cancer Intervention Study-1 (IBIS-1). This landmark study looking at chemoprevention in women at high risk of breast cancer, showed that risk reduction was only seen in those women whose MD decreased while on chemoprevention [8]. This was the first study to show that changing MD could be a potential biomarker for breast cancer risk reduction.

Following this, several studies found that a reduction in MD in women taking AET was associated with a lower risk of recurrence and death in comparison to those women whose MD did not decrease. These studies have been small, often single centre studies, with manual methods of assessing breast density and often containing a mixture of pre- and post-menopausal women. The time interval between commencement of AET and MD assessment has been variable, often within the same study. Many of the women in these studies also had chemotherapy, which is also known to decrease MD [9–15]. In 2021, a Cochrane review concluded that the evidence to support breast density change as a prognostic biomarker for treatment was of low/very low certainty. Whilst studies suggested a potentially large effect size with tamoxifen, the evidence was limited. They concluded that further research was required to assess MD as a biomarker for all classes of endocrine therapy [16]. Subsequent studies have shown promising results in the AET setting, but these studies remain small, and results were most significant in pre-menopausal women [17], women with high baseline breast density [18] or aggressive tumour characteristics [19]. Whilst the working hypothesis is that decreasing MD in women taking AET may indicate sensitivity to endocrine therapy, the exact mechanism remains unclear and is an area of active research. One theory is that decreases in density arise when a woman is able to metabolise the drug effectively [16]. There is no suggestion that decreasing MD affects survival; only that it may be a biomarker for treatment response.

Little data exists regarding the influence of MD and changes in contralateral MD on breast cancer risk in older, post-menopausal women who are treated for breast cancer. If it were possible to identify women deriving little or no therapeutic value from AET, these women would benefit from cessation of therapy and avoidance of the associated side effects and risks of such therapy [20]. The healthcare system would also gain from reduction in costs of medication and medication monitoring. Such patients may benefit from alternative AET agents [20], or therapies targeted to ER resistance pathways [21], and the efficacy of such therapies in this situation could be investigated. Conversely, women with large reductions in MD might benefit from extended AET. The optimal length of AET is currently a subject of much debate [22].

This study investigates whether the reduction in MD after 1 and/or 3 years following breast cancer diagnosis is associated with breast cancer-specific survival (BCSS), metastasis-free survival (MFS) or disease-free survival (DFS) in women over 50 years of age treated with AET who did not receive chemotherapy.

## Methods

This retrospective cohort study was generated from a subset of participants from the Mammo-50 trial [ISRCTN48534559] [23]. The Mammo-50 Trial was a prospective multicentre study (over 100 sites) of mammographic frequency in the follow-up of women aged over 50 years who had breast cancer (DCIS or invasive cancer). All Mammo-50 trial participants had a ‘normal’ year-three post-diagnosis mammogram, with no evidence of new or recurrent malignancy. Most participants had baseline diagnostic mammograms at diagnosis, and other sets from one and three years later, available for analysis on their respective hospital Picture Archiving and Communication System (PACS) for analysis. Questionnaires and documentation at recruitment to Mammo-50 and at follow-up provide details of surgical pathology, duration and type of AET, weight, height, and clinical outcomes. Patients were followed up within the Mammo-50 trial for a maximum of 6 years after randomisation (9 years post-surgery). Mammo-50 was approved by the UK Health Research Authority (HRA) (West Midlands Research Ethics Committee (REC) 13/WM/0419). Patients gave written consent for their data to be used in future research. MEDICI was also approved by the UK HRA confidentiality advisory group (CAG): 19/CAG/0149, REC: 19/WS/0112.

Participants taking AET in the absence of adjuvant chemotherapy were identified from the Mammo-50 dataset. Women aged over 50 years at breast cancer diagnosis, treated with successful breast conserving surgery (BCS), with a normal year three mammogram and relevant (diagnostic, year-one and year-three) mammograms available, were included. Exclusion criteria were bilateral breast cancer, breast cancer prior to current episode, chemotherapy, diagnosed or suspected recurrence at three years. A control group was selected from the Mammo-50 trial participants that did not take AET or have chemotherapy; this included those that were ER negative and those where ER status was not known (predominately for those with non-invasive disease).

De-identified mammographic images of the contralateral (normal) breast were retrieved from time of diagnosis (baseline) and one- and three-years post-surgery. After de-identification, images were collated for reading by experienced mammography readers.

MD was measured on the untreated breast using a 0–100% VAS. An initial batch of images from 50 patients was reviewed by eleven readers to allow assessment of inter-reader variability. All readers were trained in mammographic interpretation and reported mammograms as part of routine clinical care. Readers were either consultant radiologists or consultant or advanced practice radiographers, specialising in breast imaging. The intraclass correlation coefficient (ICC) between all 11 readers (inter-reader agreement) was 0.80 (95% confidence interval (CI): 0.77–0.84). One outlier who tended to classify MD lower than all other readers was identified. The ICC improved to 0.84 (95%CI: 0.80–0.87) when the outlier was excluded. A subset of four readers then re-read the images to allow assessment of intra-reader variability. The ICC within the four readers (intra-reader agreement) was 0.86 (95% CI 0.77–0.91). Following analysis of this pilot dataset the outlier was excluded from the full study. A further training document was compiled and supplied for the remaining readers.

Subsequently, images for all patients were batched and sent to readers at random. Two readers independently assessed density for each patient. One of these readers recorded MD of the images from all three time points. Images were presented in random order. A different reader was presented with paired images (diagnosis versus 1 year and baseline versus 3 years) and assessed whether MD had decreased, increased, or stayed the same. The degree of certainty was recorded on a five-point Likert scale: definitely decreased, probably decreased, no clear difference, probably increased and definitely increased.

### Statistical analysis

In a potential cohort of up to two thousand women treated for breast cancer with BCS and endocrine therapy but not chemotherapy, it was anticipated that approximately 33% would have a reduction in their MD and that the risk of breast cancer death would be 0.50 in comparison with those without a density reduction [10–13]. It was calculated that a hazard ratio of 2 could be detected with 90% power if 100 events were observed, and 80% power with 75 events observed.

Demographic characteristics of the participants were summarised using medians and ranges for continuous variables and frequencies for categorical variables, and compared across AET and control groups using a Wilcoxon rank sum test or Chi-squared tests as appropriate.

MD measured on a VAS was summarised at diagnosis, year 1 and year 3, using medians and interquartile ranges, for those on AET and controls. Inter-and intra- reader reliability were estimated using intra-class correlation coefficients derived from a two-way random effects model.

The percentage change in MD was calculated from diagnosis to each follow-up and compared across AET groups using a Wilcoxon rank sum test. The proportion of patients with a reduction in MD was reported for both groups and compared using a Chi-squared test. A reduction in MD based on the VAS scale was defined as 10% (or more) reduction in MD since diagnosis. This definition was chosen as the most consistent from the original International Breast Cancer Interventions 1 (IBIS 1) trial and subsequent studies [8, 13–15]. 

Frequencies for each of the responses for the reader-assessed change in MD were obtained per group and compared using a Chi-squared test. A reduction in the reader-assessed change in MD was defined as the category “definitely decreased”. A Kappa statistic was determined to assess the agreement between raters for the change in MD over time.

Initial analysis was conducted to compare the MD of those patients taking AET with those who were not (controls). It was anticipated that MD would be progressively lower in those in the AET arm, especially since some of the controls did not have ER-positive disease.

The AET arm was then divided into those who had a reduction in MD and those that did not, and differences in event rates considered. Adverse events encompassed development of locoregional recurrence or distant metastases, the clinical manifestation of treatment resistance.The primary outcome of breast cancer-specific survival (BCSS) was defined as the time from the date of trial entry until the date of death from breast cancer or censored at the date of death from other causes or date last known to be alive. Secondary outcomes of metastasis-free survival (MFS) and disease-free survival (DFS) were defined as the interval from the date of trial entry until the date of first distant recurrence or death, and the interval from the date of trial entry until the date of first recurrence, new primary or the date of death. Subgroup analysis by type of endocrine therapy and mammography equipment vendor was also performed. Kaplan-Meier survival curves for the time-to-event outcomes were constructed for the change in MD. Cox proportional hazards models were used to determine the effect of any changes in MD and to adjust for known prognostic variables. All statistical analyses were conducted within statistical analysis system (SAS) version 9.4 software.

## Results

**Fig. 1:**
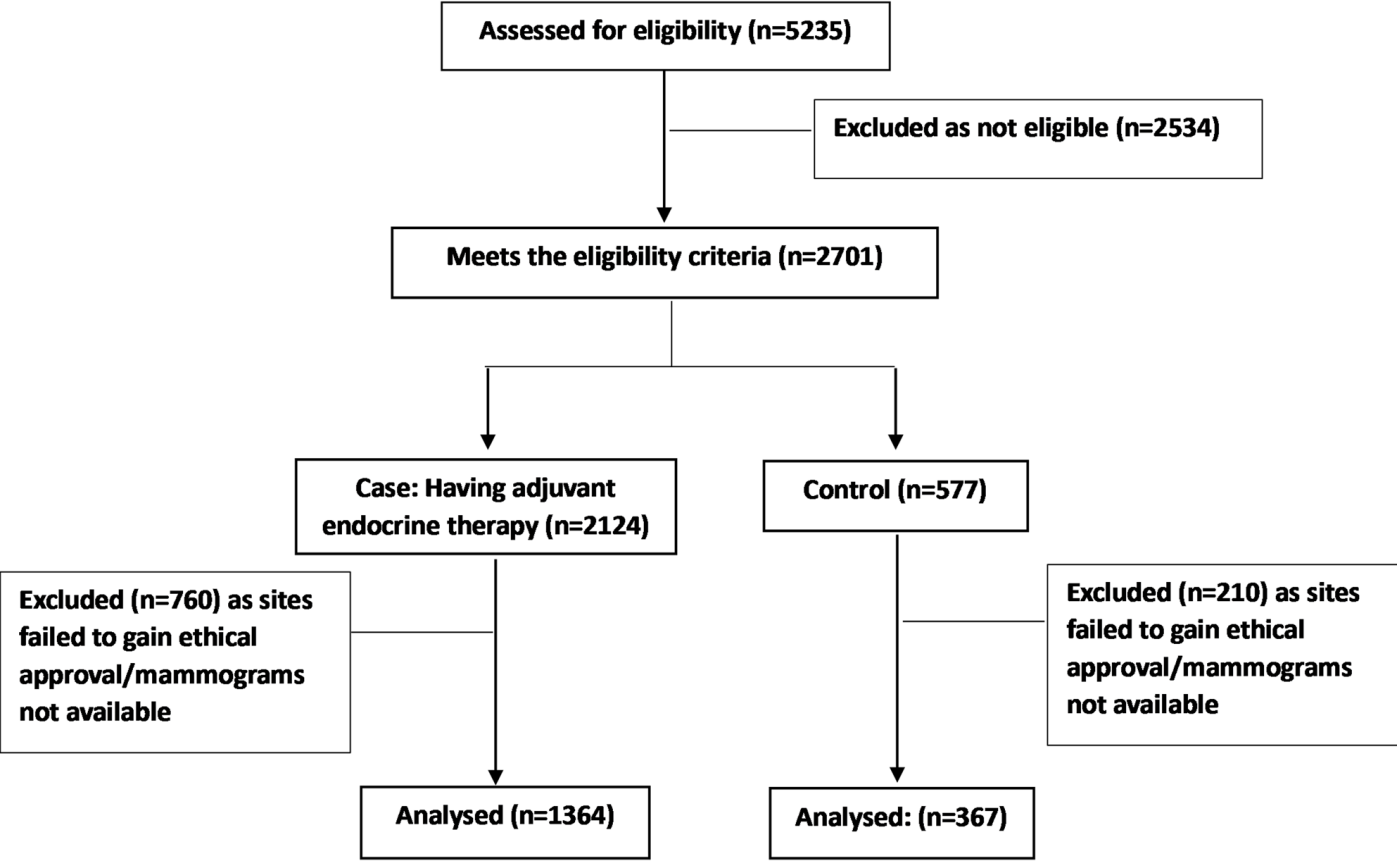
Consort diagram

Of the 2124 eligible participants who had BCS and were taking AET, 1364 (64%) participants were included; 760 participants were excluded due to lack of local approval for the MEDICI study or lack of availability of the full set of mammograms (Fig. 1). A further 367 of a possible 577 (64%) control arm participants were included. The median age of participants was older in the AET group; 67 years (range 53–91 years), versus 65 years (range 53–90 years) in the control arm, *p* = 0.0012. Almost all were post-menopausal in both the control and AET groups, 341 (93%) and 1298 (95%) respectively. The median body mass index (BMI) was 27.3 in both groups (*p* = 0.40), and the overwhelming majority of patients identified as being white (*p* = 0.32). By design, all patients in the AET group had invasive ER positive cancer. By comparison, in the control group, only 85 (23%) had invasive carcinoma, the remaining 282 (77%) had pure ductal carcinoma in situ (DCIS), and only 123 (33%) had proven ER-positive disease. Within the AET group, 385 (28%) patients were treated with tamoxifen, 787 (58%) with an aromatase inhibitor (AI) and 192 (14%) with both. Baseline characteristics are shown in Table 1.

**Table 1:** Background characteristics

	Cases (AET)	Control
Patient characteristics		
Total	1364	367
Age at trial entry		
Median (interquartile range)	67 (61–71)	65 (60–71)
Range	53–91	53–90
BMI		
Median (interquartile range)	27.3 (24.5–31.1)	27.3 (23.9–30.8)
Range	17.5–52.8	17.0-50.9
Ethnicity		
White	1334 (97.8%)	362 (98.6%)
Mixed	8 (0.6%)	0
Asian or British Asian	8 (0.6%)	4 (1.1%)
Black or Black British	7 (0.5%)	1 (0.3%)
Chinese or other	4 (0.3%)	0
Not known	3 (0.2%)	0
Tumour characteristics		
Type of disease		
DCIS	0	282 (77%)
Invasive	1364 (100%)	85 (23%)
ER status		
ER positive	1364 (100%)	123 (33%)
ER negative	0	80 (22%)
ER Not done	0	164 (45%)
Events		
All deaths	88 (6.5%)	15 (4.1%)
Breast cancer specific deaths	23 (1.7%)	6 (1.6%)
Locoregional or new breast primary	34 (2.5%)	30 (8.2%)
Any recurrence (locoregional/distant/new breast primary)	56 (4.1%)	34 (9.3%)
Distant recurrence	33 (2.4%)	10 (2.7%)
Distant metastasis or death from any cause	98 (7.2%)	18 (4.9%)
Recurrence or death from any cause	121 (8.9%)	41 (11.2%)

### Mammographic density (MD) assessment

The median MD as assessed at diagnosis with a VAS for those in the AET group was 27% (Interquartile range (IQR): 13–47%), similar to the control group (29%; IQR: 12–47%), *p* = 0.98. A total of 277 (20%) of the AET group and 75 (20%) of controls had a reduction in MD at 1 year (*p* = 0.96) and 288 (21%) of the AET group and 77 (21%) controls at 3 years (*p* = 0.93). Regarding categorical data, 200 (15%) of the AET group and 54 (15%) of the control group were considered to have a definite decrease in MD at year 1 (*p* = 0.98) and 105 (8%) and 25 (7%) respectively at year 3 (*p* = 0.56).

### Breast cancer specific survival (BCSS)

For the AET group 23 patients had died from breast cancer with a median follow-up of 8.7 years post-surgery. The 5-year BCSS rate was 99.6% (95% CI:97.4–99.9) for those with a 10% or more reduction in MD at 1 year compared to 98.3% (95% CI: 97.2–98.9) for those without a reduction in MD at 1 year (Hazard ratio [HR] = 0.56 (95% CI 0.17–1.88), *p* = 0.35. The 5-year BCSS rates were also similar for those with and without a reduction in MD on the 3-year mammogram (99.3% (95%CI:97.2–99.8) versus 98.4% (95%CI:97.4–99.0) respectively, (HR = 0.54 (95% CI 0.16–1.83; *p* = 0.32, see Table 2).

**Table 2:** Survival outcomes according to change in density as assessed by VAS

	*n*	No. events	% event free	% (95% CI) rate at	
				2 years	5 years
Breast cancer specific survival					
Reduction in MD at 1 year	HR = 0.56 (95% CI 0.17–1.88), *p* = 0.35				
10% or more reduction	277	3	98.9	100 (100–100)	99.6 (97.4–99.9)
Less than 10% reduction	1086	20	98.2	99.4 (98.8–99.8)	98.3 (97.2–98.9)
Reduction in MD at year 3	HR = 0.54 (95% CI 0.16–1.83), *p* = 0.32				
10% or more reduction	288	3	99.0	100 (100–100)	99.3 (97.2–99.8)
Less than 10% reduction	1071	19	98.2	99.5 (98.9–99.8)	98.4 (97.4–99.0)
Metastasis free survival					
Reduction in MD at 1 year	HR = 1.19 (95% CI 0.74–1.90), *p* = 0.47				
10% or more reduction	277	23	91.7	97.8 (95.2–99.0)	94.2 (90.7–96.4)
Less than 10% reduction	1086	75	93.1	98.5 (97.6–99.1)	93.6 (92.0–95.0)
Reduction in MD at year 3	HR = 1.41 (95% CI 0.91–2.21), *p* = 0.13				
10% or more reduction	288	27	90.6	97.9 (95.4–99.1)	92.6 (88.8–95.1)
Less than 10% reduction	1071	70	93.5	98.6 (97.7–99.2)	94.1 (92.5–95.4)
Disease-free survival					
Reduction in MD at 1 year	HR = 1.17 (95% CI 00.77–1.78), *p* = 0.47				
10% or more reduction	277	28	89.9	97.5 (94.8–98.8)	92.4 (88.5–94.9)
Less than 10% reduction	1086	93	91.1	98.3 (97.4–98.9)	92.6 (90.8–94.0)
Reduction in MD at year 3	HR = 1.47 (95% CI 0.99–2.19), *p* = 0.058				
10% or more reduction	288	34	88.2	97.2 (94.5–98.6)	89.7 (86.0 -93.1)
Less than 10% reduction	1071	86	92.0	98.5 (97.6–99.1)	93.4 (91.7–94.7)

Regarding categorical data, the 5-year BCSS rate was 98.3% (95%CI: 95.0-99.5) and 98.6% (95%CI 97.7–99.1) respectively for those with and without a definite reduction in MD at 1 year (HR = 0.82 (95% CI 0.24–2.77), *p* = 0.75, Table 3). BCSS was similar when considering those with and without a definite reduction 3 years post-surgery (5-year BCSS 98.9% (95% CI:92.4–99.8) versus 98.5% (95% CI:97.6–99.1) respectively, HR = 0.51 (95% CI 0.07–3.76); *p* = 0.51).

**Table 3:** Survival according to categorical change in density

	*n*		No. events		% event free		% (95% CI) rate at			
							2 years		5 years	
Breast cancer specific survival										
Change in MD from baseline to 1 year	HR = 0.82 (95% CI 0.24–2.77), *p* = 0.75									
Definitely decreased	200		3		98.5		100 (100–100)		98.3 (95.0 -99.5)	
Not definite	1164		20		98.3		99.5 (98.9–99.8)		98.6 (97.7–99.1)	
Change in MD from baseline to 3 year	HR = 0.51 (95% CI 0.07–3.76), *p* = 0.51									
Definitely decreased	105		1		99.1		100 (100–100)		98.9 (92.4–99.8)	
Not definite	1259		22		98.3		99.5 (98.9–99.8)		98.5 (97.6–99.1)	
Metastasis free survival										
Change in MD from baseline to 1 year	HR = 0.77 (95% CI 0.42–1.40), *p* = 0.39									
Definitely decreased	200		12		94.0		99.0 (96.0-99.7)		93.8 (89.4–96.5)	
Not definite	1164		86		92.6		98.3 (97.3–98.9)		93.7 (92.2–95.0)	
Change in MD from baseline to 3 year	HR = 0.70 (95% CI 0.30–1.60), *p* = 0.39									
Definitely decreased	105		6		94.3		99.0 (93.4–99.9)		95.0 (88.4–97.9)	
Not definite	1259		92		92.7		98.3 (97.4–98.9)		93.7 (92.1–94.9)	
Disease-free survival										
Change in MD from baseline to year 1	HR = 0.78 (95% CI 0.45–1.34), *p* = 0.37									
Definitely decreased	200	15		82.5		98.5 (95.4–99.5)		92.8 (88.2–95.7)		
Not definite	1164	106		90.9		98.1 (97.1–98.7)		92.5 (90.8–93.9)		
Change in MD from baseline to year 3	HR = 0.65 (95% CI 0.30–1.41), *p* = 0.28									
Definitely decreased	105	7		93.3		98.2 (97.3–98.8)		94.0 (87.2–97.3)		
Not definite	1259	114		91.0		98.2 (97.3–98.8)		92.4 (90.8–93.8)		

### Metastasis free survival (MFS)

Thirty-three patients in the AET group developed metastases in the follow-up period. The 5-year MFS rate for those with a 10% or more reduction in MD at 1 year was 94.2% (95% CI 90.7–96.4) compared to 93.6% (95% CI 92.0–95.0) for those without (HR = 1.19 (95% CI 0.74–1.90); *p* = 0.47) and 92.6% (95% CI 88.8–95.1) for those with a reduction at 3 years and 94.1% (95% CI 92.5–95.4) for those without (HR = 1.41 (95% CI 0.91–2.21); *p* = 0.13, Table 2).

Regarding the categorical data, the 5-year MFS rate for those with a definite reduction in MD at 1 year was 93.8% (95% CI:89.4–96.5) and 93.7% (95% CI:92.2–95.0) for those without (HR = 0.77 (95% CI 0.42–1.40), *p* = 0.39) and similar results were seen at 3-years post-surgery (Table 3).

### Disease free survival (DFS)

The 5-year DFS rate for those with a 10% or more reduction in MD at 1 year was 92.4% (95% CI 88.5–94.9) and 92.6% (95% CI 90.8–94.0) for those without (HR = 1.17 (95% CI 0.77–1.78); *p* = 0.47). The DFS was descriptively but not statistically worse for those with a reduction at 3 years (5-year DFS rate of 89.7% (95% CI 86.0–93.1)) compared to those without (93.4% (95% CI 91.7–94.7), HR = 1.47 (95% CI 0.99–2.19); *p* = 0.058, Table 2).

For categorical data, the 5-year DFS rate was 92.8% (95% CI 88.2–95.7) and 92.5% (95% CI 90.8–93.9) respectively for those with and without a definite reduction in MD at 1 year (HR = 0.78 (95% CI 0.45–1.34), *p* = 0.37, Table 3). The 5-year DFS rate was 94.0% (95% CI 87.2–97.3) and 92.4% (95% CI 90.8–93.8) respectively for those with and without a definite reduction in MD at 3 years, HR = 0.65 (95% CI 0.30–1.41); *p* = 0.28, (Table 3).

### Subgroup analysis according to type of endocrine therapy

Similar number of events for breast cancer specific death (6 (1.6%) vs. 13 (1.7%)), recurrence (15 (3.9%) vs. 30 (3.8%)) and metastases (8 (2.1%) vs. 21 (2.7%)) were seen between the tamoxifen and AI subgroups (see Table 4).

**Table 4:** Events according to AET

	Tamoxifen; *n* (%)	Aromatase inhibitor (AI)I; *n* (%)
Total	385	787
All deaths	25 (6.5)	52 (6.6)
Breast cancer specific deaths	6 (1.6)	13 (1.7)
Any recurrence	15 (3.9)	30 (3.8)
Metastases	8 (2.1)	21 (2.7)

For BCSS, there was no significant interaction between the type of endocrine therapy and the percentage change in MD at 1 year (*p* = 0.96) or at 3 years (*p* = 0.87). There was also no significant interaction between endocrine therapy and whether there was a definite decrease in MD at year 1 (*p* = 0.99) or at 3 years (*p* > 0.99).

Likewise, for MFS, there was no significant interaction between the type of endocrine therapy and the percentage change at year 1 (*p* = 0.46) or at 3 years (*p* = 0.38). However, there was a non-significant interaction between endocrine therapy and whether there was a definite decrease in MD at 1 year (*p* = 0.18), but those having an AI and a reported definite decrease in MD tending to have a better MFS (HR = 0.42 (95% CI 0.13–1.35)) whereas the opposite was seen for those on tamoxifen (HR 1.17 (95% CI 0.47–2.90)).

Similarly for DFS, there was no significant interaction between the type of endocrine therapy and the percentage change at 1 year (*p* = 0.71) or at 3 years (*p* = 0.79). There was also a non significant interaction between endocrine therapy and whether there was a definite decrease in MD at 1 year (*p* = 0.24) or at 3 years (*p* = 0.98).

### Subgroup analysis according to vendor

There were 1011 patients whose mammograms were acquired using the same mammography vendor throughout the 3 years; 807 of those had AET; 204 were in the control group. The three main vendors (GE, Hologic and Siemens) accounted for 238 (30%), 257 (32%) and 294 (36%) respectively.

The limited events precluded analysis to assess an interaction between the percentage change in density and BCSS according to vendor. Of the 789 women, 49 MFS events were observed. There was no significant interaction between the vendors and the percentage change at 1 year (*p* = 0.30) or at 3 years (*p* = 0.45).

## Discussion

Increasing age is a well-recognised breast cancer risk factor; however, there is also a higher prevalence of indolent oestrogen-receptor positive cancers in the older population. Thus, there is great interest in the potential for tailoring AET to those patients who will benefit and de-escalating in those cases where the benefit may be outweighed by side effects [24].

This is the largest, multicentre study to assess the potential of MD as a prognostic indicator in older breast cancer patients treated with AET in the absence of adjuvant chemotherapy following BCS. All participants in our study were aged over 50 years, with a median age of 67 years; as expected over 90% were post-menopausal [25]. In contrast to previous studies, often of a younger population, we have not identified any significant difference in any survival outcomes—BCSS, MFS or DFS—according to the presence or absence of a decrease in MD. Nor was there any significant difference in MD at any time-point between the AET and control groups, suggesting that AET had little effect on MD in this patient population. Our findings are in line with a growing body of evidence suggesting that the findings reported in studies with a higher proportion of younger and/or pre-menopausal women [10, 14, 17, 18] may not transfer to the older, post-menopausal population [9].

In a small Dutch study of 378 post-menopausal women treated with adjuvant tamoxifen and/or exemestane, MD was assessed by expert readers viewing analogue mammograms. Although 31% of included women were also treated with adjuvant chemotherapy, they reported that MD was, in general, low and did not substantially change over time, with no relationship observed between MD and the occurrence of locoregional recurrence, distant recurrence, and contralateral breast cancer [9].

Although a small Scandinavian case-control study of post-menopausal women reported a HR 0.50 (95% CI 0.27–0.93) for relative reduction in dense area (> 20% compared with ≤ 9% increase to ≤ 10% reduction) for breast cancer mortality this was only observed when MD was assessed as an absolute dense area using automated (Cumulus™) software. These findings were not replicated when percentage density was assessed [11]. Assessing absolute area is known to reduce the effect of BMI on interpretation—women with a high BMI inevitably have a greater proportion of fatty tissue—however it is not a technique available in routine clinical practice and therefore has limited practical use. A recent primary prevention study, the KARISMA trial, further supports our findings reporting that although tamoxifen at different doses effectively decreased the MD in premenopausal women, this did not occur in postmenopausal women [26].

Both Nyante and Abubakar reported a breast cancer mortality reduction associated with a decrease in MD in older Caucasian populations. However, in both of these case-control studies the majority of participants also received adjuvant chemotherapy [13, 19]. This potential confounding factor is also true of many of the other published trials reporting positive outcomes [10, 14, 18, 19]. This is important as adjuvant chemotherapy has also been shown to cause a reduction in MD [27] but its utilisation also implies more aggressive tumour characteristics. The association with decrease in MD and reduced breast cancer-specific mortality has been demonstrated to be stronger amongst women with more aggressive tumours [19].

A high pre-treatment MD has also been shown to have a significant effect on subsequent density reduction [14, 28]. The median baseline breast density in our study population was only 27% (IQR: 13–47%) suggesting that almost all patients would be considered Breast Imaging Reporting and Data System (BIRADS) category I/a or II/b density. By contrast, studies reporting a significant association between decrease in MD and survival or recurrence outcomes invariably include populations with higher average baseline MD [10, 14, 17, 18].

Notably, when subgroup analysis was performed according to type of AET (tamoxifen versus AI) in our study, there was some suggestion that decreasing MD, conferring better DFS, was more likely in the AI group. No such relationship was observed in the tamoxifen only group—despite the strongest previous evidence being for this group [16]. This may reflect poorer sex hormone suppression with tamoxifen than AIs in postmenopausal women [29].

A limiting factor of this study is that MD was subjectively assessed whereas previous studies have demonstrated stronger associations with prognostication when automated methods of absolute MD are utilised [11]. This is not, however, practical in routine UK clinical practice; and this study was designed pragmatically to replicate the clinical workflow. Additionally, density assessment was performed by single readers in the main study. To mitigate the risk of significant variation between readers inter- and intra-reader variability was assessed in the pilot study and demonstrated high correlation for both measures, greater than that deemed necessary in prior studies of MD [5]. The low pre-treatment MD may have limited the ability to detect a VAS reduction of ≥ 10% MD using VAS. Whilst this could be considered a limitation of the study, it also reflects the clinical reality for this patient population suggesting that assessing for change in MD in older post-menopausal women is unlikely to be constructive. It was postulated that observed changes in MD may be affected by the equipment and technical variations, however subgroup analysis was limited due to the low event rate. Another limitation of the study was that the control group of participants not having AET included those with negative or unknown ER status and those with DCIS to give sufficient numbers for comparison with those on AET. Thus, the differing populations may have resulted in there being no difference between the AET and control groups, although one might expect the inclusion of ER-negative controls to have accentuated differences between the groups, rather than obscuring them. The main limiting factor of this study is the low event rate. The rate of recurrences, new primaries, distant metastases and breast cancer death was lower than expected [23], and did not conform to the a-priori power calculation. This led to wide confidence intervals and as a result, a significant effect of changes in MD on outcomes cannot be excluded.

## Conclusion

Change in MD has not been shown to be a useful prognostic indicator in women over the age of 50 years whether post-menopausal or not, treated with AET in the absence of adjuvant chemotherapy. This could be related to low baseline MD, indolent disease and low event rate in this population, although these very factors also limited the power of our study to detect the target effects. The fact that we have not detected associations may, of course, reflect the true situation rather than being purely attributable to study limitations. In any event, we recommend that the lack of evidence for MD decrease as a biomarker in this group of patients should be taken into account both in clinical practice and when MD reduction is used as a surrogate outcome measure in clinical trials.

## Data Availability

The datasets used and/or analysed during the current study are available from the corresponding author on reasonable request, after publication of the primary manuscript subject to approvals. After approval a signed data sharing agreement will be required prior to data release.

## Abbreviations

AET: Adjuvant endocrine therapy
BCS: Breast conserving surgery
BCSS: Breast cancer specific survival
BIRADS: Breast imaging reporting and data system
BMI: Body mass index
CAG: Confidentiality advisory group
CI: Confidence interval
DFS: Disease free survival
HRA: Health research authority
ICC: Intraclass correlation coefficient
MD: Mammographic density
MFS: Metastasis free survival
PACS: Picture archiving and communication system
SAS: Statistical analysis system
VAS: Visual analogue scale
